# GlobDB: a comprehensive species-dereplicated microbial genome resource

**DOI:** 10.1093/bioadv/vbaf280

**Published:** 2025-11-09

**Authors:** Daan R Speth, Nick Pullen, Samuel T N Aroney, Benjamin L Coltman, Jay Osvatic, Ben J Woodcroft, Thomas Rattei, Michael Wagner

**Affiliations:** Centre for Microbiology and Environmental Systems Science, University of Vienna, 1030 Vienna, Austria; Centre for Microbiology and Environmental Systems Science, University of Vienna, 1030 Vienna, Austria; Centre for Microbiome Research, School of Biomedical Sciences, Queensland University of Technology (QUT), Translational Research Institute, Woolloongabba, QLD 4102, Australia; Centre for Microbiology and Environmental Systems Science, University of Vienna, 1030 Vienna, Austria; Joint Microbiome Facility of the Medical University of Vienna and the University of Vienna, 1030 Vienna, Austria; Department of Laboratory Medicine, Medical University of Vienna, 1090 Vienna, Austria; Centre for Microbiome Research, School of Biomedical Sciences, Queensland University of Technology (QUT), Translational Research Institute, Woolloongabba, QLD 4102, Australia; Centre for Microbiology and Environmental Systems Science, University of Vienna, 1030 Vienna, Austria; Centre for Microbiology and Environmental Systems Science, University of Vienna, 1030 Vienna, Austria; Center for Microbial Communities, Department of Chemistry and Bioscience, Aalborg University, 9220 Aalborg, Denmark

## Abstract

**Motivation:**

Over the past years, substantial numbers of microbial species’ genomes have been deposited outside of conventional INSDC databases.

**Results:**

The GlobDB aggregates 14 independent genomic catalogues to provide a comprehensive database of species-dereplicated microbial genomes, with consistent taxonomy, annotations, and additional analysis resources. The GlobDB more than doubles the number of microbial species represented by genomes relative to the field standard genome taxonomy database.

**Availability and implementation:**

The GlobDB is available at https://globdb.org/.

## 1 Introduction

Over the last twenty years, advances in DNA sequencing and data processing have led to an explosion in the available genome information of cultivated and uncultivated microorganisms. These genomes offer an unprecedented window into microbial diversity and its metabolic potential. Assembling genomes from metagenomes is now a routine practice, yielding thousands of genomes in studies focused on specific environments (Gurbich *et al.* 2023, Ma *et al.* 2023, Carlino *et al.* 2024, Chen *et al.* 2024, Cheng *et al.* 2024, Kim *et al.* 2024, Ma *et al.* 2024, Wang *et al.* 2024, Zhang *et al.* 2024, Michoud *et al.* 2025). The research community has greatly benefited from the field standard of depositing both raw and processed sequence data in International Nucleotide Sequence Database Collaboration (INSDC) databases (i.e. NCBI, ENA, and DDBJ) (Karsch-Mizrachi *et al.* 2025). Several ongoing meta-analysis efforts are processing data from many studies across environments, and have aggregated millions of microbial genomes into widely used genomic catalogues (Nayfach *et al.* 2021, Parks *et al.* 2022, Schmidt *et al.* 2023, Aroney *et al.* 2024, Dmitrijeva *et al.* 2025). While submitting unprocessed sequencing data to INSDC databases is standard practice, depositing assembled and binned metagenome recovered genomes (MAGs) of large-scale studies is less common. We have observed a recent trend that MAGs from large scale studies are deposited in generic data repositories such as Zenodo or figshare, dedicated project websites, or national databases that are not (yet) INSDC members. Lack of submission to INSDC databases leads to omission of genomes from commonly used tools such as the NCBI BLAST webserver, or INSDC-derived databases such as the genome taxonomy database (GTDB), hindering further analysis and discovery based on these data. Furthermore, the fragmented deposition of microbial genomes means that there is no longer a central resource for species-dereplicated genomes that includes the full extent of microbial diversity represented by genome sequences.

## 2 Results

### 2.1 Database description

**Figure 1.**
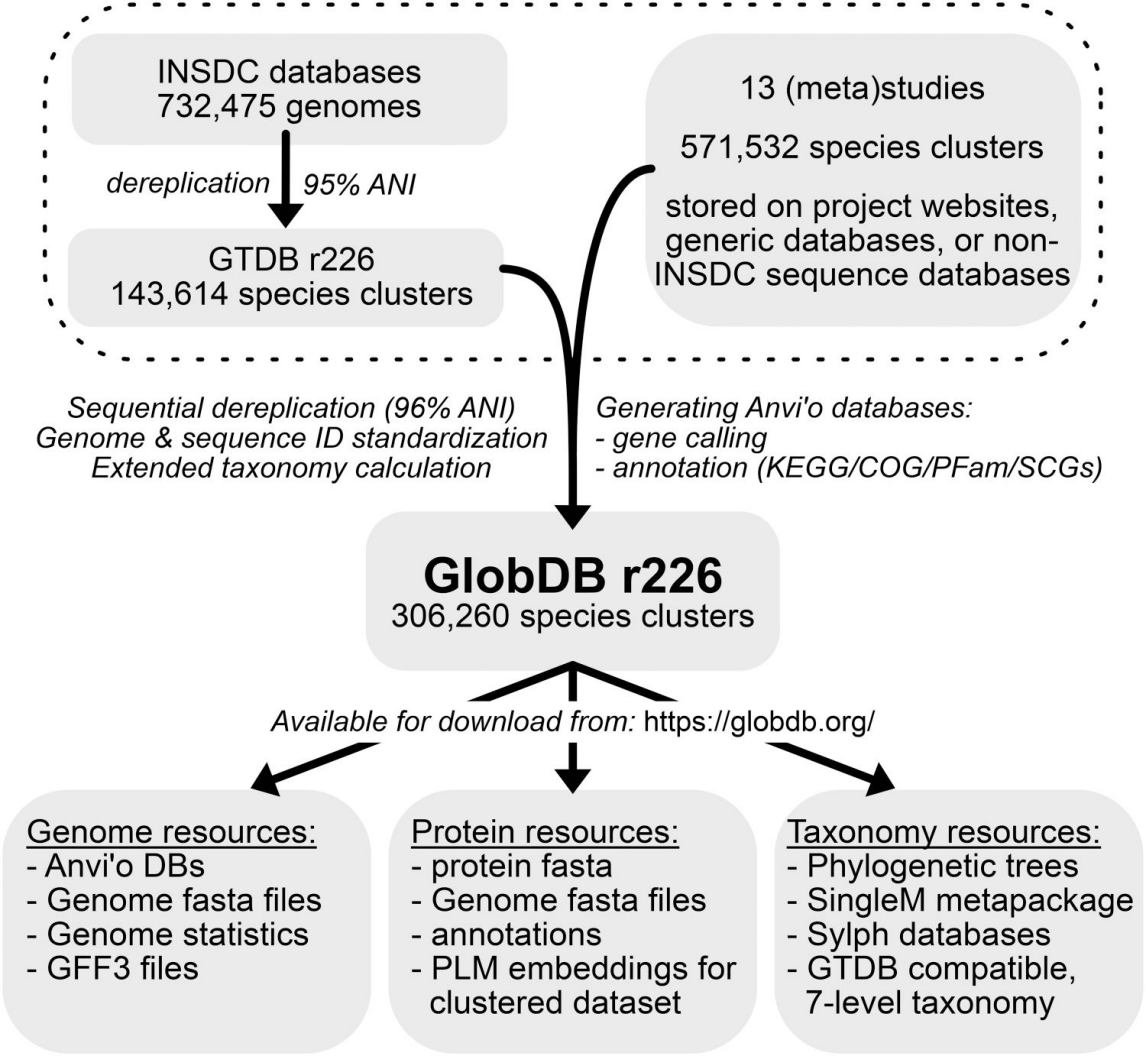
Overview of data included in, and resources available from, the GlobDB. The work indicated within the dashed box at the top of the figure indicates the source data for the GlobDB, text in italics indicates data processing, and the text within grey boxes describes resources.

We have compiled the GlobDB, a comprehensive database of species-dereplicated microbial genomes. The GlobDB aims at maximal representation of phylogenetic diversity to facilitate genome-based analysis and discovery. The GlobDB follows the annual GTDB release schedule, and as of the current release integrates 14 resources (Table 1). These were sequentially dereplicated at 96% average nucleotide identity (ANI) and 50% aligned fractions (Jain *et al.* 2018) in the order listed in Table 1. Therefore, the GTDB species representatives are the base of the GlobDB, and the other 13 datasets extend upon this. Several datasets did not provide a species-dereplicated set for download. In these cases, genomes redundant with the GlobDB were first removed, followed by dereplication of the remainder using dRep (Olm *et al.* 2017). Finally, 420 genomes were removed from the dataset due to low genome quality or putatively representing eukaryotes. The full dereplication process is described in detail on https://globdb.org/methods. The current GlobDB release 226 contains more than double the number of species-representative genomes (306 260) compared to the GTDB release 226 species representative set (143 614), indicating that >50% of known genomic species diversity is not included in INSDC databases (Fig. 1). This also leads to underrepresentation of specific environments, such as the Tibetan Plateau (TPMC) or sheep and goat guts (SHGO), in these databases (Table 1). To our knowledge, the GlobDB is the only resource that integrates large MAG collections across all environments, with a focus on bringing in MAGs that have not been included in INSDC databases and maximizing representation of microbial diversity.

**Table 1.** Resources included in the GlobDB.

Rank	Name	Number of species reps	Included in the GlobDB	Environment	Refences
1	GTDB	143 614	143 607	Global	Parks *et al.* (2022)
2	mOTU	124 295	63 092	Global	Dmitrijeva *et al.* (2025)
3	SPIRE	107 078	49 369	Global	Schmidt *et al.* (2023)
4	RBG	38 494	23 406	Global	Aroney *et al.* (2024)
5	GEM	45 599	3762	Global	Nayfach *et al.* (2021)
6	MGnify	29 556	1732	Env. specific	Gurbich *et al.* (2023)
7	GOMC	24 195	2858	Env. specific	Chen *et al.* (2024)
8	SMAG	21 078	6216	Env. specific	Ma *et al.* (2023)
9	TPMC	10 723	8644	Env. specific	Cheng *et al.* (2024)
10	KRGM	6348	334	Env. specific	Kim *et al.* (2024); Ma *et al.* (2024)
11	cFMD	10 112^a^	336	Env. specific	Carlino *et al.* (2024)
12	SHGO	5810^a^	1021	Env. specific	Zhang *et al.* (2024)
13	AMXMAG	1768^a^	506	Env. specific	Wang *et al.* (2024)
14	GFS	2862	1377	Env. specific	Michoud *et al.* (2025)

a No species-dereplicated set was available for download.

The GlobDB includes resources beyond aggregation of species representative genomes (Fig. 1). We provide standardized identifiers of each resource, and a complete 7-level taxonomy built upon and extending the GTDB taxonomy. The taxonomy of the species representatives sourced from the other 13 resources is determined using a custom approach based on the GTDB workflow (Chaumeil *et al.* 2022, Aroney *et al.* 2024), automatically assigning new taxon labels according to their Relative Evolutionary Divergence (RED) value. In practice, this results in propagating GlobDB genome identifiers upwards through the taxonomic ranks, until a node in the tree is reached that already has GTDB taxonomy assigned. This approach ensures that the GlobDB taxonomy is an extension of the GTDB taxonomy and fully compatible with it.

Sylph (Shaw and Yu 2025) databases and a SingleM metapackage (Woodcroft *et al.* 2025) of GlobDB species representative genomes are available for taxonomic profiling of metagenomes. Additional genome metadata includes: completeness, contamination, and basic genome statistics calculated using CheckM2 (Chklovski *et al.* 2023); and whether the genome accession is linked to an isolate deposited in a major culture collection assessed via BacDive (Schober *et al.* 2025).

Furthermore, anvi’o (Eren *et al.* 2021) “contigs databases” of all GlobDB genomes are made available. These SQL databases contain the genome sequences and contextual data, including: gene calls for rRNA, tRNA, and protein coding genes as well as functional annotations of protein coding genes generated using Pfam (Mistry *et al.* 2021), COG (Galperin *et al.* 2021), KEGG (Kanehisa *et al.* 2023, Kananen *et al.* 2025), and dbCAN2 CAZymes (Zhang *et al.* 2018). GFF files with gene calls and COG IDs, fastA files with amino acid sequences of protein coding genes, and tab delimited files with functional annotations of each GlobDB genome are also available for download separately.

Finally, to be able to generate protein language model (pLM) embeddings of the GlobDB proteins, we clustered the 838 615 274 GlobDB amino acid sequences using a 40% identity threshold over 80% of the length of both sequences (Steinegger and Söding 2018), yielding 82 973 016 representatives of clusters larger than one sequence. We generated ProtT5-XL-U50 (Elnaggar *et al.* 2022) pLM embeddings for 82 972 511 of these cluster representatives. Sequences, annotations, cluster membership, and pLM embeddings of this clustered sequence set are available for download. Full methods for the GlobDB can be found on https://globdb.org/methods.

In summary, we introduce the GlobDB, a comprehensive database of species-dereplicated microbial genomes and several analysis products. We expect the GlobDB to be useful in future work that benefits from a broad representation of microbial genomic diversity at any taxonomic rank. This includes studies involving phylogenomics, comparative genomics, taxonomic profiling of metagenomes, or gene and protein family analyses.

## Data Availability

The GlobDB is hosted on the Life Science Compute Cluster (LiSC) of the University of Vienna. The previous (220) and current (226) releases are available at https://globdb.org/. Currently available releases will be hosted for 10 years after publication. In addition, we intend to keep following the annual update schedule of the GTDB, and will keep previous releases available for reproducibility of work that used the GlobDB.
